# Survival Mechanisms Used by Some *Leishmania* Species to Escape Neutrophil Killing

**DOI:** 10.3389/fimmu.2017.01558

**Published:** 2017-11-16

**Authors:** Ivo B. Regli, Katiuska Passelli, Benjamin P. Hurrell, Fabienne Tacchini-Cottier

**Affiliations:** ^1^Department of Biochemistry, WHO Immunology Research and Training Collaborative Center, University of Lausanne, Lausanne, Switzerland

**Keywords:** *Leishmania*, neutrophils, *Leishmania* survival, neutrophil extracellular traps, reactive oxygen species, neutrophil granules, *Leishmania* replication

## Abstract

Neutrophils are the most abundant leukocytes in human blood. Upon microbial infection, they are massively and rapidly recruited from the circulation to sites of infection where they efficiently kill pathogens. To this end, neutrophils possess a variety of weapons that can be mobilized and become effective within hours following infection. However, several microbes including some *Leishmania* spp. have evolved a variety of mechanisms to escape neutrophil killing using these cells as a basis to better invade the host. In addition, neutrophils are also present in unhealing cutaneous lesions where their role remains to be defined. Here, we will review recent progress in the field and discuss the different strategies applied by some *Leishmania* parasites to escape from being killed by neutrophils and as recently described for *Leishmania mexicana*, even replicate within these cells. Subversion of neutrophil killing functions by *Leishmania* is a strategy that allows parasite spreading in the host with a consequent deleterious impact, transforming the primary protective role of neutrophils into a deleterious one.

## Neutrophils and *Leishmania*: A Multifaceted Story

Neglected parasitic diseases are affecting more than one million people worldwide. Amongst them, leishmaniases are a complex of diseases that affects 2 million people per year across 98 countries. The *Leishmania* protozoan parasites are transmitted by blood-sucking sand flies that deposit the parasites in the mammalian skin during their blood meal. There are more than 20 different *Leishmania* species worldwide. The infecting species together with host factors determine the various clinical manifestations leishmaniasis can have as well as the outcome of the disease. Cutaneous leishmaniasis is the most predominant form of the diseases. Following infection, an ulcerative lesion usually appears near the insect bite site. In mucocutaneous leishmaniasis, the disease affects the mucocuatenous tissues of the oro-naso-pharyngeal areas and often leads to local tissue destruction and death due to secondary infections if left untreated. Visceral leishmaniasis is characterized by hepatosplenomegaly and impeded bone marrow function due to the proliferation of parasites in macrophages within these organs. If not treated, visceral leishmaniasis patients develop cachexia, pancytopenia, subsequent immunosuppression and they eventually succumb to their disease (1). There are several treatments available against leishmaniasis of which pentavalent antimonials have been the standard of care for decades. However, these drugs have many adverse effects and the emergence of drug-resistant parasites is increasing worldwide. As the increase in drug resistance renders the available therapeutics less efficient, the need of efficient vaccines and a better understanding of the diseases is crucial to fight leishmaniases (2, 3).

Neutrophils are massively and rapidly recruited to sites of injury and microbial infections. They are the most abundant leukocytes in human blood. Neutrophils play very important roles in innate immunity and in the regulation of adaptive immune response (4, 5). They are well known for their antimicrobial functions, playing a decisive role in innate host defense against a variety of pathogens, including bacteria and fungi. To kill microbes, neutrophils possess an arsenal of weapons that include phagocytosis and subsequent microbe degradation within phagolysosomes, where granules fuse to rapidly release their microbicidal agents. Neutrophils can degranulate their granule content also in the local microenvironment and they can also kill pathogens through the production of reactive oxygen species (ROS). In addition, neutrophils can extrude neutrophil extracellular traps (NETs) that consist of a DNA backbone associated with microbicidal proteins. NETs allow entrapping of the pathogens, preventing their spread, and in some cases killing them (6). Cytokines and chemokines released by neutrophils are involved in the activation and/or recruitment of other innate cells thereby contributing to the shaping and development of an adaptive immune response (7, 8). The relevance of the role played by neutrophils in the fight against many infections is underlined by the susceptibility to repeated life-threatening bacterial and fungal infections observed in patients suffering from genetically inherited or acquired neutropenia or who have neutrophils with functional defects (9). The important role of neutrophils in regulating defense against parasites and some viruses has more recently emerged (10, 11) and increasing evidence points out to a crucial role for neutrophils in leishmaniasis disease outcome (10–13).

In contrast to their well-described protective roles in many infections, neutrophils may play a detrimental role in leishmaniasis disease development, at least in some instances. In addition to their early recruitment following infection, neutrophils were reported to infiltrate damaged tissues of human mucosal leishmaniasis (14) and to be present in the chronic form of the disease in human and animals (14–20). Following experimental infection with most *Leishmania* spp. neutrophils are rapidly and massively recruited to the site of parasite inoculation where they rapidly phagocytose most of the parasites present. Several groups have used genetically neutropenic mice or mice rendered neutropenic by injection of anti-neutrophil antibodies to show the importance of this early wave of neutrophil on disease outcome. Collectively, most of these studies reported that neutropenic mice had a better disease outcome, indicating a negative role for neutrophils in some forms of cutaneous leishmaniasis (2, 21–24). In contrast, neutrophils may facilitate parasite clearance as observed for *Leishmania braziliensis* and *Leishmania amazonensis* (25–30) and for *Leishmania donovani* (31). However, *L. amazonensis* killing appeared to be parasite stage-dependent as promastigotes, the infecting form of the parasites, but not amastigotes, the intracellular replicative forms of the parasite, were killed *in vitro* by neutrophils (32).

One of the immune evasion strategies used by *Leishmania* parasites may be linked to the status of neutrophil apoptosis as phagocytosis of apoptotic neutrophils has been shown to impair dendritic cells (DCs) maturation and the development of an efficient adaptive immune response [reviewed in Ref. (7)]. Indeed, internalization of apoptotic *Leishmania major*-infected neutrophils by DCs impaired development of *Leishmania*-specific immune response (33, 34). Interaction of apoptotic neutrophils with macrophages also has a negative impact on the disease (35). Following *Leishmania* delivery by sand fly bite or needle inoculation, parasites were reported to induce, delay or have no impact on neutrophil apoptosis, depending on the *Leishmania* spp. or the origin of neutrophils. *Leishmania mexicana* did not influence dermal neutrophil survival *ex vivo* (36) and *L. infantum* did not induce neutrophil apoptosis *in vitro* (37). In contrast, *L. brasiliensis* induced neutrophil apoptosis, at least *in vitro* (30). *L. major* infection induced murine neutrophil apoptosis in the dermis (22, 34) while it delayed human blood-derived neutrophil apoptosis (22, 34, 38, 39). These results suggest that the effect of *Leishmania* on neutrophil apoptosis may differ between murine and human neutrophils, or the difference observed may come from the diverse *Leishmania* spp. or neutrophil origins.

Recent data reported that a subset of low density neutrophils expressing HLA-DR express high levels of PDL1 in human CL and VL patients (19, 40), a marker promoting T cell exhaustion. These data suggest a novel negative role for this neutrophil subset in leishmaniasis.

## The Distinct Mechanisms Used by *Leishmania* spp. to Escape Killing by Neutrophils

*Leishmania* are using neutrophils transiently to finally be ingested by macrophages, their final host. The parasites may be released by dying neutrophils and/or infected apoptotic neutrophils may be phagocytosed by macrophages. This latter process referred to as the “Trojan horse” entry in macrophages, confers a silent entry for the parasites in these cells (41). We will now discuss the several mechanisms used by some *Leishmania* spp. to escape neutrophil killing and even in some cases how the parasites can use these cells to replicate, collectively resulting in a negative impact on disease outcome.

**Figure 1:**
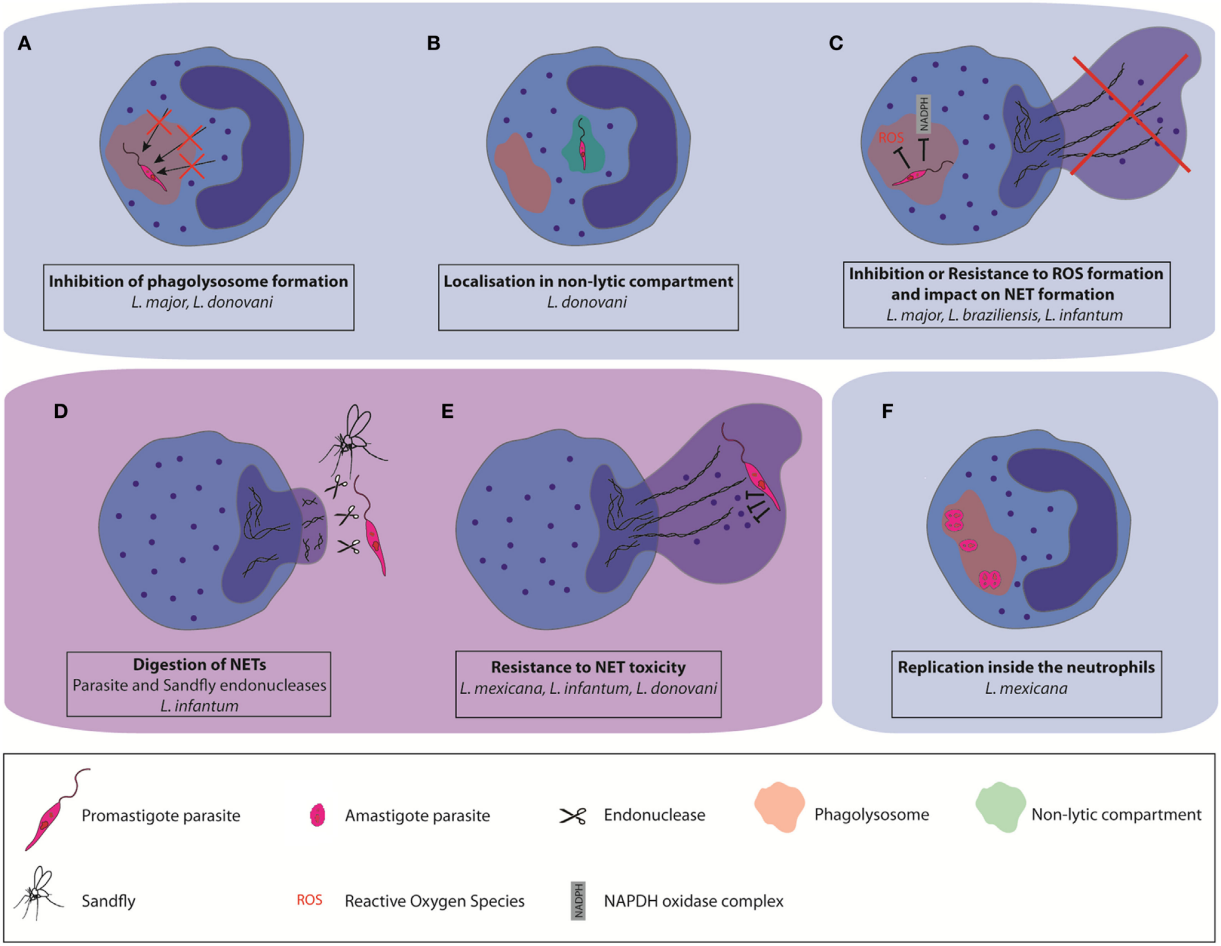
Different mechanisms used by some *Leishmania* spp. to escape neutrophil killing. *Leishmania* can impair parasite destruction by neutrophils **(A)** by affecting the formation of mature phagolysosomes and their fusion with neutrophil granules, **(B)** by localization in non-lytic compartments, and **(C)** by resisting to the toxicity associated with reactive oxygen species production. Some *Leishmania* spp. can also resist to the microbicity associated with neutrophil extracellular trap (NET) formation **(D)** by directly inhibiting NET formation, or by digestion of the NET scaffold using pathogen-or vector-derived endonucleases **(E)**. They can also resist NET antimicrobial factors through the expression of protease-resistant surface molecules. **(F)** A subset of *L. mexicana* amastigotes was shown to replicate in neutrophils.

Using *in vivo* two-photon imaging, intact and live parasites have been detected in neutrophils during the first days of *L. major* and *L. mexicana* infections, revealing that a good proportion of parasites can resist neutrophil microbicidal functions (22, 36). Several strategies used by *Leishmania* parasites to escape killing by neutrophils have been described. During neutrophil development there is a continuity of granule formation, including azurophil granules (primary or peroxidase-positive granules), specific (secondary granules), and gelatinase granules (tertiary granules). Secretory granules are formed last (42). During the maturation of myeloblasts into neutrophils, more than 300 different proteins are stored into granules. One of the ways parasites may survive in neutrophils is through interference in the process of granule fusion with the *Leishmania* containing phagosome. *In vitro* studies showed that *L. major* and *L. donovani* promastigotes regulate granule fusion with phagosomes, allowing azurophil but preventing specific and gelatinase granule fusion with parasite-containing phagosomes (43). This prevents their destruction by neutrophils microbicidal granule contents (Figure 1A). In addition, *L. donovani* was shown to traffic to non-lytic compartments within neutrophils (44), establishing yet another strategy to escape the neutrophil killing machinery (Figure 1B).

In addition to the release of antimicrobial molecules, the assembly of a functional NADPH oxidase (NOX2) is playing a crucial role for neutrophil microbicidal function (45). NOX2 assembly is inducing the generation of reactive oxygen species (ROS), a process called oxidative burst. Interference with oxidative burst increases pathogen survival within neutrophils. It has been shown that *L. major* does not elicit the generation of ROS upon phagocytosis by human neutrophils (Figure 1C) (43). However, *L. braziliensis* induce high levels of ROS production upon infection of human and murine neutrophils but ROS generation in human neutrophils did not affect parasite survival (31, 45). In addition to its major role in neutrophil intracellular killing functions, NOX2-mediated generation of ROS has also been reported to be crucial for classical (NADPH-dependent) NET formation. This is exemplified by the lack of NET formation in patients with chronic granulomatous disease (46, 47) and restoration of NET formation in these patients upon re-introduction of NOX2 by genetic engineering (48). Moreover, there also exists ROS-independent NET release. *L. amazonensis* promastigotes were shown to elicit both types of NETs and be killed by them (29). Thus, the impact of parasites on ROS formation is also *Leishmania* spp. dependent.

## Pathogens Escape from NETs

Upon activation, neutrophils can form NETs that can entrap and often kill pathogens, reviewed in Ref. (49). However, several microbes including some *Leishmania* spp. have developed various mechanisms to escape NET trapping and/or killing. Whether parasites are killed or not by NETs depends on the involved *Leishmania* spp. For instance, in humans, *L. amazonensis* was shown to induce NET formation and to be killed by them (50). In contrast, NETs failed to kill (36) *L. infantum* (51) *and L. donovani* (52) parasites. Furthermore, murine NETs were not able to kill *L. mexicana* (36).

A very efficient strategy used by *Leishmania infantum* (51) is to prevent NET formation by suppressing or inducing decreased efficiency of the oxidative burst (Figure 1C).

As another strategy to avoid NET killing, several microbes express nucleases that degrade the NET DNA backbone. For example, surface DNAse and wall anchored nuclease expression were reported in Gram-positive bacteria (53–56) and for several Gram-negative bacteria (53, 57–59). NET degrading endonucleases have also been reported in Gram-negative bacteria (60–62). Expression of the enzyme 3′nucleotidase/nuclease by *Leishmania* also contributes to protection from the microbicidal activity of NETs as shown for *L. infantum* (51). In addition, the parasite sand fly vector may interfere with NET formation. The saliva of the New World *Leishmania* vector, *Lutzomyia longipalpis*, was shown to contain an endonuclease capable of degrading NETs (63). As salivary gland proteins are deposited by the sandfly in the host during the insect blood meal its endonucleases may indirectly influence the role of NETs in the disease pathogenesis (Figure 1D).

Microbes may also avoid NET killing through the synthesis of cell surface components rendering them resistant to NET-associated protease activity (Figure 1E). This has been observed for *L. amazonensis* and *L. donovani. Leishmania* surface coat is densely packed with lipophosphoglycan (LPG), a glycoconjugate that is polymorphic among *Leishmania* spp. and which is differentially expressed in the infective promastigote form compared to the replicative amastigote form (64). In *L. amazonensis*, LPG was shown to induce NET formation, and confer resistance to NET-mediated killing by forming a thick glycocalyx that protects the parasite from microbicidal agents (50). In contrast, LPG of *L. donovani*, was shown not to induce NET formation, although it also conferred protection against NET mediated parasite killing (52). Peripheral blood neutrophils from active VL patients were unable to release NETs despite an active phenotype (65), showing that the replicating amastigote stage of the parasites also has an impact on neutrophil functions, contributing to the pathology of the disease.

## Neutrophils as a Place to Replicate

Neutrophils are short-lived non-dividing cells that become rapidly apoptotic in the circulation. However, during inflammation and infection, the neutrophil lifespan can be extended to several days (66), although it still remains difficult to estimate neutrophil lifespan in tissues, mostly due to technical issues. For some *Leishmania* spp. transient inhibition or delay of neutrophil apoptosis is an obvious strategy to allow prolongation of their presence within these cells. The PI3K/AKT, ERK1/2 p28MAPK pathways which maintain expression of the antiapoptotic Mcl1 protein were shown to contribute to prolonged neutrophil lifespan in *L. major* infection (67).

The induction of delayed neutrophil apoptosis together with the inhibition of neutrophil killing machinery elicited by some *Leishmania* spp. suggested that the parasite could use these cells to replicate. *Leishmania* parasites have two life cycle stages, the infective flagellated promastigote form which is elongated with a size comprised between 6 and 12 µm, not including the flagellum length, and the replicative, non-flagellated amastigote form, which is intracellular and of smaller size (3–5 µm). The sand fly is depositing in the skin metacyclic promastigotes, a process inducing rapid recruitment of neutrophils. It is therefore not surprising that most studies investigating interactions between neutrophils and *Leishmania* have been performed with the promastigote form of the parasite, reviewed in Ref. (12, 13, 24). In addition, neutrophils have been detected in smears of unhealing cutaneous lesions of *L. braziliensis* patients, at a time when the parasite is in its intracellular amastigote form. The presence of neutrophil-attracting chemokine mRNA was observed in biopsies of patients with chronic lesions due to *L. panamensis* and *L. braziliensis*, suggesting neutrophil presence in the lesion. Also, neutrophils were observed in biopsies of tegumentary leishmaniasis patients (14, 68–71). Furthermore, neutrophil presence was also observed in unhealing lesions of experimental cutaneous leishmaniasis following *L. major* (18, 21) and *L. mexicana* infection (36). Very few studies have investigated the interactions between neutrophils and the amastigote form of the parasite. The group of Soong was the first to show that neutrophils internalized *in vitro L. amazonensis* and *L. braziliensis* amastigotes. While *L. amazonensis* amastigotes survived in neutrophils, *L. braziliensis* amastigotes were efficiently killed (28, 32). We recently reported that *L. mexicana* amastigotes are also internalized and survive in neutrophils *in vitro*. After overnight incubation, we observed an average of one amastigote per neutrophils. In contrast, the majority of lesion-derived neutrophils harbored >2 intact amastigotes per neutrophil. Imaging of the lesion-derived neutrophils showed the presence of several aligned amastigotes within neutrophils, suggesting possible parasite replication in these cells. Parasite uptake by neutrophils was relatively neutral, eliciting low level of apoptosis or neutrophil activation in infected neutrophils (20). To measure parasite replication, we generated transgenic parasites expressing a photoconvertible GFP mKikume gene (72). These *L. mex*^SWITCH^ parasites express constitutively green fluorescence that can be converted to red fluorescence upon exposure to a pulse of violet light. Upon cell division, the photoconverted red proteins are diluted as *de novo* green protein in synthesized, and the fluorescence recovery after conversion (FRAC) is measured in dividing cells. Analysis of FRAC by imaging flow cytometry and time-lapse microscopy revealed that, 48 h after photoconversion, a subset of highly infected neutrophils containing more than 4 amastigotes per cell showed high replication (Figure 1F). Amastigotes were found in large vesicular acidic compartment. In macrophages, *Leishmania* amastigotes reside in phagolysosome-like compartments called parasitophorous vacuoles (PVs) where they multiply. For most *Leishmania* spp. one amastigote is enclosed within these PVs with little vacuole space. However, *L. mexicana* and *L. amazonensis* form upon division communal large PVs containing numerous amastigotes, a process diluting toxic components and directly linked to parasite evasion to host immune responses (73, 74). We observed larger Lysosensor-positive vacuoles in *L. mexicana* infected neutrophils (20), suggesting the formation of communal PVs in neutrophils. It remains to be determined whether the replication of amastigotes in neutrophils is linked to the presence of these large PVs.

The majority of parasite replication is taking place in macrophages, and most lesional parasites divide at a slow rate even if, as observed *in vitro*, there is likely variability in the growth rates of parasites in unhealing cutaneous lesions (75). Indeed, in a recent study a small subset of parasites that appeared to divide rapidly was reported. These parasites could use neutrophils as a safe transient place to replicate.

The demonstration that a subset of *L. mexicana* parasites is able to replicate within neutrophils revealed a novel role of neutrophils that can act as a niche for parasite replication during the chronic phase of infection. However, there very likely exist differences in the ability of the invading *Leishmania* spp. to replicate in neutrophils. These could originate from parasite factors but also from host factors.

## Concluding Remarks

The primary function of neutrophils in innate immunity resides in killing invading microorganisms. It is therefore not surprising that some pathogens have evolved several ways to escape elimination by these cells, allowing their silent entry in the host and even sometimes their replication in these cells. Caution in the interpretation of some of these studies should be taken as most human studies are performed with peripheral blood neutrophils that functionally differ from extravasated neutrophils present in inflamed tissues. To better understand the relevance of neutrophil functions *in vivo*, experimental murine models are used. However, it should be kept in mind that functional differences exist between mouse and human neutrophils as well, including differences in the antimicrobial repertoire and number of circulating neutrophils (76). That being said, the generation of new tools such as two-photon microscopy imaging (77) and the use of photo-switchable pathogens (78) for probing pathogen biology during infections should allow finer investigation of the mechanisms used by pathogens to promote their own survival in neutrophils *in vivo*. Furthermore, neutrophils appear to be a more heterogeneous cell population than previously anticipated (79) and new markers defining mature from immature circulating neutrophils are emerging (80). It will thus be interesting to assess whether selective *Leishmania* spp. transient survival and/or replication occur in a specific neutrophil subset, while *Leishmania* killing would take place in other subsets.

Survival of pathogens in neutrophils is not specific to *Leishmania*, indeed several bacteria, fungi or viruses are also able to escape neutrophil killing and use these cells to propagate in the host, reviewed in Ref. (81). For instance, intracellular bacteria including *Francisella tularensis* (82), *Neisseira gonorrhoae* (83), *Chlamydia pneumonia* (84); and more recently, *Yersina* spp. (85) have been shown to replicate *in vitro* in human or murine neutrophils, suggesting that not only *Leishmania* parasites but also other pathogens are diverting the primary neutrophil killing function to their own benefit and dissemination in the invaded host. Finer understanding of the mechanisms used by some *Leishmania* spp. to block neutrophil effector functions will be important in the design of prophylactic or therapeutic measures taken against leishmaniasis.

## Author Contributions

IR and FTC wrote the review. BH, KP, and IR contributed to the figures. All authors provided input to the review.

## Conflict of Interest Statement

The authors declare that the research was conducted in the absence of any commercial or financial relationships that could be construed as a potential conflict of interest.
